# Hospital and Institutional News

**Published:** 1913-03-15

**Authors:** 


### HOSPITAL AND INSTITUTIONAL NEWS.

### THE LESSON OF THE IRISH HOSPITAL DISPUTE.

It is not necessary to go into elaborate details of the dispute between the visiting and consulting staff of the Royal National Hospital for Consumption, Newcastle, Co. Wicklow, and the Board, to see where the ultimate blame must lie. Briefly, the facts may be summarised as follows. In April 1911 the two visiting physicians, having been invited by the Board to express their views on a proposal to invite another medical man to try a special treatment, reported against it. Later the Board invited the resident and consulting staffs to consider the desirability of adopting that or any other special treatment. Thereupon the staff unanimously replied that medical treatment was solely the province of the medical staff, and that methods unproved by scientific research should not be adopted. In December 1912 the medical staff learnt that the Board had appointed the outside medical man already mentioned an additional visiting physician, on the ground that, the patients having grown since 1896 from 24 to 100, the visiting staff needed augmenting. The latter and the resident staff, taking this to mean that a method of treatment of which they disapproved would be adopted, tendered their resignation. However, a conference followed between four representatives of the staff and of the Board, at which a draft agreement was approved to the effect that the staff would withdraw their resignations if the *status quo* before the additional appointment were restored, and if two of their members should be elected annually to the Board of Management. The staff further agreed to approve the appointment of the medical man above mentioned as pathologist, under the direction of the visiting physicians. The Board, however, has rejected the draft agreement, and the resignations of the consulting and visiting staffs are the result.

### NO CHANNEL OF INTERCOMMUNICATION.

The latter's circular letter to the profession, referred to last week, is regarded by the Board as an attempt to boycott the institution, which, however, Mr. John R. Orpen, the hon. secretary, states in effect to be an impossibility. When we add that the Board at present contains no representative of the medical staff, for the two recent resignations reduced their gradually diminishing number to zero, it will be plain to every man of administrative experience, whether medical or lay, that the present disastrous situation is the inevitable result of excluding the medical staff from the Board of Management. The whole dispute shows how utterly evil are the effects of such exclusion on medical and administrative officers alike. If there had been a systematic channel of intercommunication between the two the medical staff would never have had to complain of any usurpation of their functions; and the Board would never have felt that the medical staff was too small and too conservative. Such friction is simply the result of want of intercommunication. The best proof of this is the fact that at the only time when a properly formal conference took place between both parties the dispute was within an ace of amicable settlement. If the Board and the staff do not know how best to correlate their functions we may refer them to the very instructive papers and discussions which were devoted at the British Hospitals Association Birmingham Conference last autumn to this very point. They were published practically in full in THE HOSPITAL of September 8 and October 5, 1912.

### A FURTHER VICTIM OF SANATORIUM BENEFIT.

The inadequacy of the so-called Sanatorium Benefit under the National Insurance Act is again illustrated by the following case. A patient in North London in the incipient but severe stage of tuberculosis was recommended—such are the authenticated details already published—by her panel doctor sanatorium or tuberculin treatment. On applying to the Middlesex Insurance Committee, domiciliary treatment was decided on, although the Committee had never even seen the patient. Rather than let such callousness result in her death, another doctor gained her admission into a chest hospital. Comment surely is needless, for to meet the case it would probably be libellous, and what possible use could there be in libelling an Act of Parliament which is first badly drawn, and then so confused by the subsequent regulations for its administration as to force every member on the Committees thus created to choose between throwing over the rules he was appointed to administer, or allowing the rules to result in the death of those whom they were supposed to "benefit"? The inhumanity of men is a negligible quantity compared with that of the systems which human carelessness or ignorance may create.

### UNSUCCESSFUL ACTION AGAINST A SURGEON.

The action brought at Birmingham by Mr. Thomas Pollock, tube manufacturer, against Dr. Charles Leedham-Green for alleged unskilful treatment has resulted in a verdict for the defendant. It appears from the evidence that, after consulting Dr. Leedham-Green in April 1911 about the removal of a small wart from her left thumb, Mrs. Pollock went into a nursing home. Put briefly, the wart was removed and Mrs. Pollock said she afterwards suffered great pain, and that eventually the thumb had to be removed. Dr. Leedham-Green, who denied that he had bandaged the forearm, as alleged, with rubber bandages previous to the operation, stated that he had used a local anæsthetic, and that he could not find any recorded instance of a disturbance from the anæsthetic that he used. Mrs. Pollock, he said, suffered from cold hands and feet, which alone would cause gangrene at times. The jury returned a verdict for the defendant. While congratulating Dr. Leedham-Green on this verdict, we trust the case will remind surgeons always to regard the medical aspect of a patient's circulation, so as to guard against any symptom of Raynaud's Disease or other vaso-motor disturbance.

### THE TOOTING BEC EXTENSION SCHEME.

An interesting scheme for the enlargement of Tooting Bec Mental Hospital was approved by the managers of the Metropolitan Asylums District last Saturday. It had been prepared by Mr. T. W. Aldwinckle, architect, who estimated the cost of the immediate scheme at £88,000, of which £15,000 is for administrative purposes, and the remainder for patients' accommodation. At present the Board's accommodation for unimprovable imbeciles is fully occupied, while this class of patient is increasing in numbers. Last July, it may be recalled, the Board purchased the Bushey Down Estate, adjoining the Tooting Bec institution, and here it is proposed to build two men's and two women's pavilions, having 610 beds in all. The completed scheme, which would cost probably £150,000, contemplates eventually seven pavilions with a total accommodation for 1,103 patients. The problem presented by the apparent increase in the number of London's imbeciles is therefore to be dealt with on a large scale by one institution.

### FURTHER FRUITS OF PANEL SERVITUDE.

At an inquest at Woolwich last week on a carman named Emsden, it was stated that the deceased, feeling very ill, went to see his panel doctor, Dr. Andrew Mair, of Plumstead, but found the surgery so much crowded with waiting people that he returned home to bed. His daughter then went to ask the doctor to come as soon as possible, but by the time when he arrived the man was dead. Dr. Mair said that the man ought to have been under treatment for some time, but that had the man rung the bell he would have been attended to. "My patients," he added, "have increased in number since the Insurance Act began to work." A juryman suggested that something should be done to relieve the pressure on panel doctors, in which the Coroner agreed. But we can tell them what the "something" should be—namely, to restore the promised but repudiated right to free choice of doctor. Every life which has been lost so far through the pressure of the institution of panel servitude is undoubtedly part of the price which is exacted from the insured by those who have repudiated their solemn promises to maintain free choice of doctor. The insured, in fact, may be first deprived of their own doctor and then made to run the risk of dying through the want of proper attention which the burden of panel servitude entails. Such is the cost—to those who are not politicians—of party legislation. In the case of Emsden, who was stated to have died of chronic pleurisy and fatty degeneration of the heart, it remains an open question whether or not, had he been attended to, his life would have been saved. But this is only another way of showing that the panel system is the worst way possible of attempting to save it. Why does not our distinguished contemporary, the *New Witness*, whose campaign against the Insurance Act has been so courageously and ably conducted by Mr. Cecil Chesterton, not pay more attention than it has of late to the evils of the panel system? No doubt Mr. Chesterton has his hands rather full at present, but a glance at our columns every week is enough to convince anyone that the panel servitude with medical "benefit" is the heel of Achilles in the Insurance Act, on which the best-informed critics of the Act must continue to fasten. There is no lack of material for criticism or examples of the Act's attendant evils here.

### THE INSURANCE ACT AND WATERLOO HOSPITAL.

Mr. J. Topham Richardson, treasurer of the Royal Waterloo Hospital, stated at the annual meeting held last Monday that the support given to the hospital by Workmen's Benevolent Associations already showed a distinct falling off. He added that he greatly feared that the Insurance Act would affect the support which the institution had hitherto received from King Edward's Fund and the Hospital Saturday Fund. Mr. Topham Richardson is one of the first to include the King's Fund in the list of those whose aid is thought likely to be diminished by the disastrous Insurance Act.

### A "MODEL" FORM FOR CONTRACTING OUT.

At a meeting of the London Insurance Committee last week, presided over by Mr. J. A. Dawes, M.P., the General Purposes Sub-Committee reported that Mr. E. B. Turner and Dr. Evan Jones had been elected by the medical practitioners to serve on the Committee; and that the Commissioners had appointed Dr. Lauriston Shaw, Mrs. Florence Willey, and Dr. H. H. Mills as members of it. But the most interesting report received was that in which the Medical Benefit Sub-Committee stated that they had under consideration a model form (43/1.c.) which had been received from the Commissioners "for use by persons wishing to make their own arrangements for medical attendance and treatment." The attitude of the Insurance Committees towards medical benefit we referred to a fortnight ago. But the three points in the Medical Benefit Sub-Committee's report—namely, the "model" form for use by persons desiring to contract out, the recognition that the applications of special classes to contract out demand and may receive favourable consideration, and that these insured persons who desire a special treatment may be allowed to obtain it by contracting out—deserve the most careful attention. The fact that such parade is made of consideration for those who "may desire" to make their private arrangements proves again, if that be necessary, that the "right" to free choice of doctor which was solemnly promised again and again has been deliberately filched from them; and what is more, that the right itself is not only a moral one—which only the inhumanity of supposed party interests would attempt to withhold—but in innumerable instances a sheer necessity of the case. The percentage of insured sick persons who are requiring special treatment, especially when that is naively defined, as here, to be "treatment not undertaken by doctors on the panel," is very large; so are those classes of persons whose circumstances demand exemption. In short, a more eloquent testimony to the practical evils resulting from the scandalous repudiation of the promised right to free choice of doctor cannot be found than in the report of the Medical Benefit Sub-Committee. These evils are destined to grow, and if the wide existing opposition to a medical "benefit" without free choice of doctor cannot win the right to free choice by itself, the evils to patients under panel servitude should be quite enough to secure it.

### THE INSURED AWAY FROM HOME.

It is not very astonishing that the conference recently held at the offices of the Insurance Commission on the provision of medical attendance for insured persons temporarily away from home failed to provide any solution of so difficult a problem. Medical men were present from holiday resorts, wherein the difficulty is especially felt; as well as from industrial centres, from which crowds of insured holiday-makers resort to the seaside. The strictly mathematical way of equalising matters, and of providing the insured with panel attendance while away from home, would be to deduct from the panel doctor's *per capita* remuneration a proportion corresponding to the length of each person's holiday, and to utilise the money in paying the panel doctor in health resorts. This, however, would involve so much record keeping and trouble that it is impracticable; and even apart from that, it would fail to be just or satisfactory. It has to be considered that the holiday-maker, as a rule, is healthy when he goes away, and not very likely to fall ill while enjoying himself at a seaside or other pleasure resort; and the problem of convalescents complicates matters more still. Indeed, the entire question is so thorny and difficult that we doubt whether any workable solution of it is possible, short of doing injustice to some one—that is, to the insured, to their panel doctors at home, or to the panel doctors at the health resorts.

### ROUTINE FRICTION FOR WANT OF "RAPPORT."

For neglecting to forward certificates to the vaccination officer to the Kingston Board of Guardians, Mr. W. D. Elsam, the public vaccinator of the borough, Dr. James Donald, of 31 St. James Street, Kingston, has been fined, including costs, £7 16s. at Kingston Police Court. From the proceedings it appeared that every month Mr. Elsam sent a list of the unvaccinated children to the public vaccinator, whose duty it was to wait on the parents, offer to vaccinate the children, and in the case of successful vaccination or postponement to send a certificate to that effect. If Mr. Elsam did not receive any certificate he sent a final warning notice to the parent, and then took proceedings for penalties. In the six cases before the Court the parents had complied with the law, but the defendant had not forwarded the certificates. Dr. Donald's defence was that of a busy professional man who had been in the habit of waiting till the certificates accumulated, so as to avoid the inconvenience of a variety of individual postings. An outbreak of broncho-pneumonia, however, said counsel, had temporarily put a stop to vaccinations, so the certificates accumulated more slowly than usual. The moral of this appears to be that it is the want of system rather than want of public spirit in either party which has led to the trouble. Certificates must be sent in, but there could surely be sufficient *rapport* between two officials of the same borough to get over all inconvenience in what is simply a small but necessary matter of routine.

### SUGGESTED SALARIES FOR VACCINATION OFFICERS.

Owing to the diminution in the number of people vaccinated the fees of public vaccinators have fallen off considerably during the last few years, the result being that Boards of Guardians are continually being asked by the public vaccinators to increase the fees paid to them. The consent of the Local Government Board has recently been requested by the Lambeth Board of Guardians to sanction an increase and to pay public vaccinators a fee for each conscientious objection to vaccination received by them. The Local Government Board, replying to the Guardians, suggest that if a further increase of fees payable to the officers did not appear desirable, the Guardians should consider remunerating their vaccination officers by fixed salaries. The Board would be willing to assent to a departure from the terms of the Vaccination Order to enable this to be done.

### THE IMPERSONATION OF DEAD PRACTITIONERS.

For a layman to act the part of a medical man is generally regarded as too difficult to tempt anyone in need of augmenting his income to undertake the risk. Though only a romantic layman would entertain the hope of augmenting his income by such a financially precarious profession as medicine. Still, the difficulty, the requisite address. and the romantic layman are sometimes to be found together as was the case last week, when Judge Lumley Smith sentenced Harry Virtue, otherwise Richard Henry Barber, L.R.C.P., to nine months' imprisonment in the second division for giving false death certificates in which he appeared as Mr. Barber, L.R.C.P. It seemed that the late Mr. Barber was known to the prisoner, and had been accidentally drowned in 1904, after having been in practice at Portland, Oregon. The General Medical Council was unaware of his death, and in 1906 and at subsequent dates received requests, now proved to be in the prisoner's handwriting, for changing Dr. Barber's address to Liverpool and elsewhere. The prisoner was born at Manchester, and after working as both grocer and butcher went to the United States, where apparently he studied veterinary surgery, and in 1904 became assistant to a London veterinary surgeon by representing himself as another man. There was, said counsel for the prosecution, evidence that he performed operations on people. For the defence it was urged that on four occasions he had been re-engaged as *locum tenens* and had received forty testimonials, "had studied medicine at Chicago, and veterinary surgery," and so could not be called quite without experience. The sentence was, of course, inevitable, but "Dr." Virtue seems to have a certain cleverness, which, here as elsewhere, is not always associated with the quality of his name, and the medical profession may accept his testimonials with a genial smile and take them philosophically as probably quite genuine, and, if so, an interesting tribute to the art, if hardly to the science, of medicine.

### THE SALE OF POISONS.

Some interesting details concerning the administration of the penal clauses of the Pharmacy Acts are contained in the Annual Report of the Registrar of the Pharmaceutical Society, which was presented to the Council of the Society at their recent sitting. It is one of the duties of the Council to investigate cases of the alleged irregular sale of poisons, and some idea of the amount of work involved can be obtained from the fact that last year over thirteen hundred such cases were investigated. It was only found necessary to institute legal proceedings in 238 cases, in none of which was the Society unsuccessful. The majority of the offenders were unqualified drug-store proprietors, but there was also a number of chemists among the defendants. In several cases of alleged infringement of the law relating to the sale of poisons it was found on investigation that the poison asked for had been omitted from the article sold—for instance, paregoric was sold containing no opium. By this means the sellers evaded the Pharmacy Acts, but at the same time rendered themselves liable to penalties under the Merchandise Marks Act, and in several cases the Society obtained convictions under the latter Act.

### THE QUEEN AND PATIENTS' LIBRARIES.

Some months ago we had the pleasure to record a gift of books from the King to the patients' libraries of one or two London hospitals. It is now announced that the Queen has sent a parcel of books to the library at the Crippled Boys' Home, Kensington. As we pointed out on the former occasion patients' libraries have often competed with the mortuary unit for the doubtful honour of being the most neglected department of modern institutions, which pride themselves, often quite justly, of being up to date in every other respect. As good journalism consists in ramming home neglected truths by constant reiteration, we make no apology for repeating that patients' libraries should not be limited, as they often are, to stale magazines and works of piety. The first work for the library of the Crippled Boys' Home at Kensington to procure, as it probably has long ago realised, is Stevenson's "Treasure Island," the second "Swiss Family Robinson," and the third Dumas' "Three Musketeers." With this hint we may leave the subject, thankful for the encouragement which the Queen has given to institutional libraries everywhere by her quiet present of books, which should serve to remind the authorities of the Crippled Boys' Home of their duty in this matter, and to encourage every other institution to give that short half-hour's intelligent care and attention to the subject, which is all that it need demand from a committee sufficiently alert to grasp the moral as well as to accept the substance of her Majesty's gift.

### HUNGER-STRIKERS IN OLD BRIDEWELL.

Sir George Savage has done good service to the Government and the public—neither of which, in spite of the experience which institutional workers and the medical profession have placed at their disposal, has yet escaped from a cowardly mental muddle over the proper attitude to adopt towards hunger-strikers—by quoting from Lieut.-Colonel Alf. J. Copeland's "Bridewell Royal Hospital" the following record:—"Edward Coppinger having wilfully abstained from meat, and otherwise tormented himself, died in Bridewell, July 16, 1591." On this question the institutional world must be credited with all the honours—namely, practical experience, medical knowledge, and, thanks to Lieut.-Colonel Copeland and Sir George Savage, the public expression of both. As we have previously pointed out, the Government's prestige has fallen so low, thanks to the disgraceful muddling of the Insurance Act and to the still more disgraceful chicaneries which have been its outcome, that it has not sufficient *morale* to face the music even of those sentimentalists who would raise their little pipe if the prisoners who refused their food were not prevented from taking the consequences. Though how a lay public with an ounce of common sense (if such exist) could quarrel with a Government which provided good food for its prisoners, but refused to be entangled in their destiny beyond seeing that the supply was regular and wholesome, we cannot understand. Institutional workers have far too long an experience of "hunger-strikers" to take romantic views of this matter, and in consequence have invented the alternative of artificial feeding, preceded, when necessary, by the administration of sedatives, if, as occurs in mental hospitals, the patient is likely to injure himself or herself by struggling with his feeders. For it is the struggling, not the artificial feeding, which causes exhaustion and may undermine health. This way being too simple for the Government, the institutional world has generously provided a further proposal, which we should like to see discussed in our columns. Its author was a medical man who recently suggested in the lay press that if milk were substituted for water the recalcitrance of the hunger-strikers would be overcome, because he had never heard of a "hunger-thirst" being attempted, and believed that it could not be carried out, so overpowering is the demand of the human organism for liquid. Here, at least, is the only reasonable alternative we have heard to the method adopted at old Bridewell. Can our readers throw further light on the experience which led to its being forbidden?

### LORD TREDEGAR AND NEWPORT HOSPITAL.

Last Tuesday Lord Tredegar died at Newport at the advanced age of eighty-two. Known to the public at large as the Crimean veteran who led a squadron in the charge of the Light Brigade, as a landowner and a generous supporter of charities, he was an institutional worker as well. It was he, for example, who gave the site for the County Hospital at Newport, of which he was the active president. The new hospital was built between 1897 and 1901, and in 1909 he received the freedom of the borough in consideration of his services to the hospital and to other institutions, indeed to the general civic life of Newport. He is succeeded in the barony and the baronetcy by his nephew, Lieut.-Colonel Courtenay Evan. The viscounty becomes extinct. The best monument to his work, and the engaging personality which was its instrument, is probably the many reminiscences of him which the innumerable societies and charities with which he was associated possess. Though perhaps at his best before an agricultural audience—for he was a landowner first of all—his speeches were often witty, and always direct. No charity with which he came into contact was untouched by his personality, which survives in a literary sense by a collection of his anecdotes and *obiter dicta* which was published in 1911.

### STAFF VACANCIES AT ST. BARTHOLOMEW'S.

Not long ago we drew attention to the very unusual rate of vacancies and promotions on the surgical staff at St. Bartholomew's Hospital during recent years. On the medical side, however, promotion has stagnated dreadfully, but it would seem that the physicians' turn has come at last. About a year ago Dr. Horder was appointed junior assistant physician after a weary wait of years; and now it is announced that actually two vacancies are to be filled together on the senior medical staff, due to the retirement of Dr. J. A. Ormerod and Dr. Samuel West. This entails a strong probability that there will be in due course two vacancies for assistant physicians. It is so long since the younger Bart.'s physicians had chances of promotion (except last year, when Dr. Horder's claims were admittedly quite exceptional) that there will doubtless be keen competition for these two posts. It is also announced that the office of physician-accoucheur is vacant at St. Bartholomew's; this is owing to the retirement of Sir Francis Champneys, and will entail a vacancy for an assistant in that department if Dr. Griffith and Dr. Williamson are promoted. Sir Francis, we may note, was on the visiting staff at St. George's about twenty-five years ago, but transferred his labours to Bart.'s, a most unusual step nowadays. With every respect for interesting links with the past, we think that the time has come when St. Bartholomew's might abandon the time-honoured title of physician-accoucheur in favour of some other which shall designate more accurately the functions pertaining to the appointment. Why not gynæcologist, gynæcological surgeon, or obstetric surgeon?

### HIGH MORTALITY AND WORKHOUSE NURSERIES.

The question of the mortality rate of infants in workhouse nurseries has often engaged the attention of medical officers and of those Guardians who have, not always discreetly, shown a sense of responsibility for the welfare of workhouse children generally. The Poor-Law Commission's reports had certain things to say in this connection, but the facts in relation to the true difficulties of the problem are not generally understood outside medical and administrative circles. In a recent letter to the lay press a writer has reminded the public that the Children Act gave the oversight of these unwanted babies, who are placed out to nurse, to the Guardians, and there is little doubt that one of the Act's intentions was to mitigate the evils of neglect and bad feeding, from which this class of infant suffers more probably than any other. It is a question which we should like to have discussed by infirmary superintendents: whether the system of putting out infants to nurse cannot be abolished? For in the case of infants not yet weaned the expert supervision of the medical officer is essential, and how can that be gained in practice except when the infants are wholly under his control, and in the proper surroundings provided by the workhouse nursery, which is, or should be in fact, only the children's ward of every workhouse infirmary? The substitution of another system to that of putting out to nurse presents obvious difficulties. We believe, however, that medical officers and infirmary superintendents would have little difficulty in suggesting their solution, and for this vitally important purpose we invite their expressions of opinion, and their experience of acting upon them.

### A SURGICAL APPOINTMENT GOING BEGGING.

It is not only at St. Bartholomew's that vacancies are under discussion. The Royal Free Hospital wants an assistant surgeon in place of Mr. Roughton, whose health has unfortunately completely broken down. The Kent and Canterbury Hospital requires a visiting surgeon too; and the Bristol General Hospital will appoint either a physician or a surgeon to take charge of the throat department there. In the latter case the indifference of the managers is somewhat curious, for throat specialism is well recognised as the province of surgery, not of medicine. Lastly, St. Paul's Hospital for Skin and Genito-Urinary Diseases, Red Lion Square, still lacks the assistant surgeon for whom an advertisement has been appearing for some months. It is not often that a London hospital appointment goes begging as long as this one has done.

### AN EXTRAORDINARY RUN OF LEGACIES.

Only a fortnight ago we had occasion to note the good fortune of some of the Glasgow hospitals recently in the matter of substantial legacies. It never rains but it pours, and certainly Glasgow's "luck is in" just now. Last Saturday the trustees of the late Mr. Edward Davis announced the distribution on their discretionary of funds amounting, after payment of legacy duty, to over £53,000. The trustees' choice was restricted by the testator to general hospitals or infirmaries in Scotland. Glasgow gets £40,000 out of the total, the Royal Infirmary and the Western Infirmary receiving £15,000 apiece, and the Victoria Infirmary £10,000. The Edinburgh Royal Infirmary takes £2,000, and the Infirmaries at Aberdeen, Dundee, Inverness, Perth, and Dumfries £1,000 each. The County Hospital, Ayr, comes next with £750; Stirling, Greenock, Paisley, and Kilmarnock £500 each; Oban and Helensburgh £300 each. It is also announced that a further sum will eventually become available, but that no applications with regard to it can at present be entertained. Mr. Davis was a resident at Cheltenham at the time of his decease, and presumably the executors knew of some desire on his part specially to favour Glasgow charities.

### THE NORTHERN HOSPITAL TURNED SANATORIUM.

Having gained the approval of the Local Government Board, the managers of the Metropolitan Asylums Board have arranged for some 200 tuberculosis patients to be received and treated under the Insurance Act in certain pavilions allocated for the purpose in the Northern Hospital. Dr. J. E. Squire, senior physician to Mount Vernon Hospital (if that institution survives the annual meeting of its governors this week), and consulting physician to the Benenden Sanatorium, who was recently appointed visiting consultant at the Downs Sanatorium, has been selected for similar work at the Northern Hospital until March 1914, the remuneration being at the rate of 200 guineas per annum. We trust that this appointment is a sign of grace on the part of the Metropolitan Asylums Board, that they have taken to heart our repeated warnings of the necessity for appointing officers of special experience for the treatment and also for the administrative control of any tuberculosis patients who may be handed over to them. Over a visiting consultant there could be no excuse for going wrong. Let us hope that similar care will be taken over any appointments which they may make to the still more important resident posts.

### RAISING THE SALARIES OF MEDICAL OFFICERS

The Surrey County Medical Officer, Dr. T. Henry Jones, has had his salary raised from 800*l*. to 1,000*l*. a year, not only in view of the increased work which has fallen to his share of late years, but as a recognition of the satisfaction which he caused in the discharge of his duties.

### NEW SCHEME FOR TRAINING TUBERCULOSIS OFFICERS.

A strong, but temperate, warning has been issued to the governors of the Mount Vernon Hospital for Diseases of the Chest, in the form of a letter signed by Dr. S. H. Habershon and other elected representatives of the Brompton, Victoria Park, Royal Chest, Mount Vernon, and Ventnor Hospitals, urging them to postpone any decision to sell the hospital at Hampstead. The warning preceded by a few days the annual meeting, which was held on Wednesday last. Its authors point to the fact that the Mount Vernon Hospital is one of the few existing chest hospitals for the necessitous, and for the training of practitioners in the detection and management of diseases of the chest. They remark that no time could be worse than the present for considering such a proposal, since a comprehensive scheme for the training of tuberculosis officers is engaging the present attention of the Local Government Board, the other chest hospitals, and the Commissioners. In addition they remark that the present position of the chest hospitals in relation to sanatorium benefit in London is still *sub judice*. Finally, they urge that the number of patients which can be dealt with in open-air sanatoria is very limited, and that the need for such central institutions as the Mount Vernon for the diagnosis and treatment of *all* forms of chest disease has never been so great as it is to-day. The writers suggest, as an alternative to an immediate decision on the question of selling the hospital, a conference between representatives of the governors, committees, and medical staffs of the chest hospitals with the public bodies engaged in the so-called anti-phthisis campaign before any further action be taken. As we go to press the annual meeting is being held.

### THE WARDS AT COLNEY HATCH MENTAL HOSPITAL.

For some time past work has been in progress at Colney Hatch Mental Hospital for the improvement of the wards by bringing them up to the standard of modern requirements, especially as regards lighting and ventilation and the plastering of walls. So far an expenditure of £14,000 has been voted by the London County Council for these improvements. It is proposed to undertake another section of the work during the coming financial year, the wards to be dealt with being Nos. 1 to 6 on the male side. The total cost of this section of the work is estimated at £1,900. The improvements proposed are similar to those which have been carried out in other wards: the removal of the iron frames and the provision of wooden sashes and frames, the plastering of internal walls, the addition of borrowed lights and screens, and of picture rails.

### THIS WEEK'S DRUG MARKET.

The advancing tendency in the price of cod-liver oil continues, and, unless the results of the fishing improves considerably, there is every possibility that the price will advance to double the figure quoted last summer. It might not be inadvisable to buy at an early date. Citric acid also continues to advance as a result of shortage of raw materials. Opium is receding in price, and makers of codeine have announced a reduction in their quotations. The price of olive oil is well maintained. The position of quinine remains unchanged. Carbolic acid tends lower in price. At the recent public sales in Mincing Lane good prices were obtained for ipecacuanha, and sarsaparilla sold at higher prices.

